# *In vitro* investigation of electrospun PVDF-TrFE fiber mats to reduce connective tissue growth after cochlear implantation

**DOI:** 10.3389/fbioe.2026.1826304

**Published:** 2026-05-26

**Authors:** Vinzent Braemer, Jan Drexler, Lisa Kötter, Marc Mueller, Thomas Lenarz, Birgit Glasmacher, Gerrit Paasche

**Affiliations:** 1 Department of Otorhinolaryngology, Head and Neck Surgery, Hannover Medical School, Hannover, Germany; 2 Lower Saxony Center for Biomedical Engineering, Implant Research and Development, Hannover, Germany; 3 Institute for Multiphase Processes, Leibniz University Hannover, Garbsen, Germany; 4 Hearing4all, Hannover Medical School, Hannover, Germany

**Keywords:** cell-material response, cochlear implant, electrical conductivity, electrode surface modification, electrospinning, PVDF-TrFE fiber mats

## Abstract

**Introduction:**

Cochlear implant electrodes are frequently ensheathed by connective tissue as a consequence of surgical trauma and foreign body response, which can impair electrical stimulation. In this study, fiber mats fabricated by electrospinning with three different spinning times (15, 30 and 45 min) were evaluated for their suitability as surface modifications for cochlear implant electrodes to reduce connective tissue-induced insulation after implantation.

**Methods:**

Their biocompatibility was assessed through incubation with an extract solution generated from the fiber mat using two different cell types and methods. The influence of the three different polymeric structures on electrical conductivity was examined by coating SEM-holders and measuring changes in impedance. In addition, the wetting behavior of the fiber mats was monitored over a period of 3 weeks. Cell proliferation was further investigated by directly seeding NIH/3T3 eGFP fibroblasts onto the fiber mats and analyzing their growth in a period of 7 days.

**Results and Discussion:**

The fiber mats were considered biocompatible, although incubation with 100% extract resulted in decreased cell viability in NIH/3T3 fibroblasts. Impedance values increased with increasing fiber mat thickness. Wetting behavior was independent of mat thickness, occurring primarily within the first 24 h and reaching a plateau after 1–2 weeks of immersion in physiological sodium chloride solution. Although cell proliferation was reduced on the fiber mats compared to the conventional cell culture substrate after the 7 days of incubation, cell growth was delayed rather than completely inhibited. Overall, surface modification of cochlear implant electrodes with electrospun fiber mats shows potential to mitigate electrode insulation caused by connective tissue formation.

## 1 Introduction

Patients with severe to profound hearing loss are commonly treated with cochlear implants (CI), which electrically stimulate residual auditory neurons to restore hearing. Implant performance is strongly correlated to changes in the electrical interface between the electrode array and the surrounding cochlea (Newbold et al., 2011). Following implantation, insertional trauma and the foreign body response trigger the formation of connective tissue around the electrode array within the first few weeks after surgery (Newbold et al., 2004). This additional sheath increases electrical impedance, reduces charge transfer efficiency and consequently limits CI performance (Newbold et al., 2014). Moreover, the extent of electrode array encapsulation varies between patients, leading to variable increases in impedance (Somdas et al., 2007).

The vertebrate inner ear contains a diverse range of cell types, including primary sensory neurons known as spiral ganglion neurons (SGNs), which are located within the Rosenthal’s canal of the modiolus (Mattotti et al., 2015; Nist-Lund et al., 2022). SGNs are responsible for transmitting auditory signals from the inner ear to the central nervous system and are frequently used in ototoxicity studies (Yamahara et al., 2019; Schwieger et al., 2025). Consequently, they are well suited for biocompatibility testing of materials intended for CI surface modifications.

Surface modifications to either promote or inhibit cell adhesion and proliferation have been widely investigated (Zreiqat et al., 2005; Zuchowska et al., 2016; Zuppolini et al., 2020; Wulf et al., 2022). Zuchowska et al. (2016) reported reduced proliferation of human fetal lung fibroblasts (MRC-5) and human lung carcinoma cells (A549) on unmodified poly(dimethylsiloxane) (PDMS) lab-on-a-chip systems, whereas PDMS surfaces modified with poly-L-lysine or collagen enhanced cell proliferation. Similar observations were reported by Zuppolini et al. (2020) using human mesenchymal stem cells (hMSCs) on electrospun poly(ε-caprolactone) (PCL) fiber mat scaffolds. Unmodified PCL fiber mats reduced cell adhesion and viability, while polydopamine (PDA) coated fibers increased cell adhesion.

For CI applications, previous approaches involving electrode coatings with poly-L-lactic acid (PLLA) (Wulf et al., 2022), dexamethasone/PCL (Luo et al., 2021) or sodium hyaluronate hydrogel (CHA) (Xu et al., 2018) or nano structuring platinum electrodes with a femtosecond laser (Linke et al., 2015) showed promising results in reducing inflammatory reactions and impedance increases. However, they were not investigated *in vivo* regarding their long-term effect (Xu et al., 2018; Wulf et al., 2022) or failed to provide a long-term solution (Linke et al., 2015; Luo et al., 2021) delaying connective tissue growth but not preventing it.

Electrospinning as an electrohydrodynamic process used to generate thin fibers offers a further method to modify CI electrode surfaces, as shown in earlier studies (Dencker et al., 2017). The resulting fine, porous micro-networks can reduce cell adhesion, thereby minimizing the foreign body response and the associated electrical insulation of the implant. Poly(vinylidene fluoride-co-trifluoroethylene) (PVDF-TrFE) is a biocompatible polymer widely used in biomedical applications. The piezo-electric property of this material has been used in electroactive membranes (Park et al., 2018; Slobodian et al., 2022), nanogenerators (Chen et al., 2017) or even guiding scaffolds for cell adhesion, proliferation and alignment (Orkwis et al., 2020). Additionally, the inherent hydrophobicity of PVDF-TrFE, combined with the micro-network created through electrospinning, may further reduce cell attachment and thus minimize implant insulation. Nevertheless, the additional layer of fiber mat is also likely to affect the electrical properties of the electrode.

In the current study, fiber mats consisting of PVDF-TrFE are investigated *in vitro* regarding their general potential to serve as a surface modification for CI stimulation electrodes *in vivo* by examining electric conductivity, biocompatibility, and growth of fibroblasts on their surface. A detailed in depth analysis of all effects seen is not part of this first study but has to be addressed in succeeding investigations.

## 2 Methods

### 2.1 Electrospinning and morphological examination

Solutions for electrospinning were prepared according to the protocol of Gryshkov et al. by dissolving 20% w/w PVDF-TrFE (Piezotech FC30 Arkema, Düsseldorf, Germany) in a 60:40 (v/v) mixture of dimethylformamide (DMF; ≥99.5%, Carl Roth, Karlsruhe, Germany) and acetone (≥99.5%, Carl Roth) (Gryshkov et al., 2021). The solutions were homogenized at 60 °C and 100 rpm for at least 18 h using a heatable magnetic stirrer (MR3001; Heidolph, Schwabach, Germany). Electrospinning was performed in an in-house-built vertical top-down setup with a 20 cm cannula-to-collector distance. A high voltage of −23 kV [AU-30N1-LC(220V)]; Matsusada Precision Inc., Kusatsu, Japan) was applied to the cannula (21G Sterican; B. Braun, Melsungen, Germany). The polymer solution was delivered to the cannula using a syringe pump (Fusion Touch 200, Chemyx, Stafford, United States) at a flow rate of 3 mL/h. Fibers were collected without alignment on a grounded drum collector (diameter 10 cm) covered with aluminium foil to facilitate removal and handling of the fiber mats. The drum rotated with a surface velocity of 1.57 m/s. Spinning times of 15, 30, and 45 min were used to produce fiber mats of different thicknesses. Ambient conditions were in the range of 17.4 °C–20.1 °C for temperature and 30%–69% for relative humidity. Scanning electron microscopy (S-3400N, Hitachi, Tokyo, Japan) was used to examine the morphology of the electrospun fibers. All samples were sputtered for 40 s using a gold target prior to examination (JFC-1300, Jeol, Tokyo, Japan). The fiber diameter and thickness of the fiber mats were evaluated image-based (AxioVision V 4.9.1.0, Carl Zeiss, Oberkochen, Germany) for all spinning durations.

### 2.2 Biocompatibility testing

#### 2.2.1 Extract solution

To examine the cell response to the polymeric structure, an extract was prepared by incubating the fiber mat in cell specific culture medium (Dulbecco’s Modified Eagle’s Medium (DMEM) (Bio&Sell, Berlin, Germany) for fibroblasts and supplemented Panserin 401 (Pan Biotech, Passau, Germany) for SGNs. Therefore, rectangular samples measuring 12 cm^2^ (2 × 6 cm) were prepared from the fiber mat of 30 min spinning time and sterilized by washing them once in 70% ethanol and twice in phosphate buffered saline solution (PBS; Invitrogen, Karlsruhe, Germany). Under sterile conditions, the fiber mats were then dried for 4 or 24 h to allow evaporation of residual ethanol.

After the drying period, the samples were placed in 10 mL DURAN glass flasks (Th. Geyer GmbH & Co. KG, Renningen, Germany) and immersed in 2 mL of cell-specific culture medium, following the ratio defined in EN ISO 10993–12 (6 cm^2^/mL) (International Organization for Standardization, 2021). Finally, the flasks were shaken at 300 rpm and 37 °C in a MaxQ 6000 (Thermo Scientific, Darmstadt, Germany) incubator for 24 h, before the extracts were directly used for the cytotoxicity tests. In addition, one reference flask containing only 2 mL of culture medium was placed in the incubator alongside the experimental flasks and analyzed to exclude any process-related effects on the cell culture medium.

#### 2.2.2 NIH/3T3 fibroblasts and MTT-test

NIH/3T3 eGFP mouse fibroblasts were cultured in DMEM supplemented with 10% fetal calf serum (FCS) (Bio&Sell, Berlin, Germany) at 37 °C and 5% CO_2_.

For the viability test, 10,000 cells/well were seeded into a 96-multiwell culture plate (Nunc A/S, Roskilde, Denmark) and incubated at 37 °C and 5% CO_2_.

After 24 h of cultivation, the culture medium was removed and replaced with fiber mat extract solutions diluted in supplemented DMEM at eight different concentrations (100%–0.78%). Each concentration was tested in triplicates, and the experiment was repeated six times for both drying conditions (4 h and 24 h). For each plate, supplemented DMEM without exposure to the fiber mat was added in triplicates on both ends of the experimental groups and served as positive control. Moreover, a 1:1 dimethyl sulfoxide (DMSO) (Sigma Aldrich, Taufkirchen, Germany) DMEM solution served as negative control and was examined in triplicates on every plate.

Prior to its addition, the 3-(4,5-dimethylthiazol-2-yl)-2,5-diphenyltetrazolium bromide (MTT) was dissolved in DMEM without phenol red (1 mg/mL) and sterilized by filtration through a PES membrane filter unit (pore size = 0.22 µm; Merck Millipore Ltd., Carrigtwohill, Ireland). Following 24 h of exposure to the extracts, the supernatant of each well was aspirated and 50 µL of the MTT solution was added. The plate was then incubated for 2 h at 37 °C. The formation of formazan crystals was stopped by decantation, and 100 µL of 2-isopropanol (Sigma Aldrich) was pipetted into each well to dissolve the crystals. By placing the plate in a microplate Synergy H1 Hybrid Reader (BioTek, Waldbronn, Germany) and shaking it orbitally at 180 rpm for 10 min, complete dissolution of the formazan crystals was ensured. Finally, the absorbance was measured at a wavelength of 570 nm to quantify cell viability in each well. Plates with mean differences of more than 15% between the positive controls on each plate were excluded from the results according to DIN EN ISO 10993-5 (International Organization for Standardization, 2009). Cell viability was calculated by normalizing the values for mean absorbance of each experimental group on each plate to the respective positive control.

#### 2.2.3 Spiral ganglion neurons

In a second approach, the biocompatibility of the fiber mats was evaluated by incubating primary spiral ganglion neurons (SGNs) with the prepared extracts and assessing their survival and neurite outgrowth.

All experiments including primary cells were conducted in accordance with the German “Law on Protecting Animals” (§4) and the European Directive 2010/63/EU for the protection of animals used for experimental purposes. The procedures were approved and registered with the local authorities (registration no. 2023/251). Early postnatal Sprague Dawley rats (4–5 days old) of both sexes from an in-house colony were used for spiral ganglion cells (SGC) preparation and primary cell culture. All experimental procedures were carried out and reported in compliance with institutional and national regulations.

Decapitation and further dissection of the cranial cavity was performed as previously described (Schwieger et al., 2015). Briefly, the cochlea was removed from the dissected skull halves and further processed under a reflected light stereomicroscope (MZ6; Leica Biosystems, Wetzlar, Germany) to obtain the SGC.

For enzymatic dissociation of the SGC, the cooled Panserin 401 was replaced with pre-heated Panserin 401 enriched with 0.1% trypsin and 0.01% DNase I (Roche, Mannheim, Germany) and incubated for 15 min at 37 °C and 5% CO_2_. Intermediate shaking helped the dissociation process. After the incubation, the majority of the digestion solution was aspirated and 200 µL of preheated FCS was added to stop the enzymatic activity. Subsequently, the cell cluster was washed three times with 1 mL of complemented Panserin 401 containing Insulin (8.7 μg/mL; Biochrom, Berlin, Germany), Penicillin (30 U/mL; Biochrom), Glucose (0.15%; B. Braun), PBS (0.172 mg/mL), HEPES (2-(4-(2-hydroxyethyl)-1-piperazinyl)ethanesulfonic acid) buffer solution (23.43 µM; Invitrogen) and N2-supplement (0.1 μL/mL; Invitrogen).

Complete dissociation of the SGC was ensured by mechanically resuspending the cells in 1 mL of supplemented Panserin 401 with two different types of pipettes (1,000 µL & 200 µL) until no visible cell cluster remained. The cell number was determined by exclusion test with trypan blue and a Neubauer chamber. SGC were seeded at a density of 1 × 10^4^ cells/well into 96-multiwell culture plates coated with poly-D/L-ornithine (0.1 mg/mL; Sigma Aldrich) and naturel mouse laminin (0.01 mg/mL; Invitrogen).

The next steps - including incubation, medium change and exposure of the cells to the extract solution - were conducted identically to the MTT assay. Control wells used to determine the seeding density were fixated with a 1:1 acetone/methanol solution 4 h after seeding, whereas experimental wells were fixated after 24 h of exposure to the different concentrations of extract solution (48 h cultivation time in total).

For the quantification of neuronal survival and measurement of the neurite length, immunocytochemical labelling was performed as described by Wefstaedt et al. (2005). Summarized concisely, a monoclonal mouse anti-200 kDa neurofilament antibody (NovacastraTM; Leica) served as the primary antibody, followed by detection with the Vectastain® Elite ABC Kit (Vector Laboratories, California, United States) and final visualization by staining using a DAB peroxidase substrate kit (Vector Laboratories). Each experiment was independently performed five times, with triplicates on every plate.

Under a transmitted light microscope (CKX41SF; Olympus, Hamburg, Germany), stained neurons exhibiting neurite outgrowth at least three times their soma diameter were counted for each well and normalized to the positive control (complemented Panserin 401 solution without contact to the fiber mat). Secondly, neurite length was determined by following the protocol described by Gillespie et al. (Gillespie et al., 2001). Briefly, each well was divided into five sections of equal size and the three neurons with the longest neurites (15 neurons per well) were measured using the polygon function of an image analysis program (cellSens, Olympus). For wells containing less than 15 surviving neurons, all neurons were included in the analysis regardless of their position within the well. The average neurite length for each experimental group was then calculated and normalized to the positive control.

### 2.3 Electrical conductivity measurements

Coating the stimulation electrodes with an additional layer of non-conductive material increases their electrical resistance. Hence, impedance measurements were conducted in this study to evaluate the influence of the fiber mat on electrical conductivity.

**FIGURE 1:**
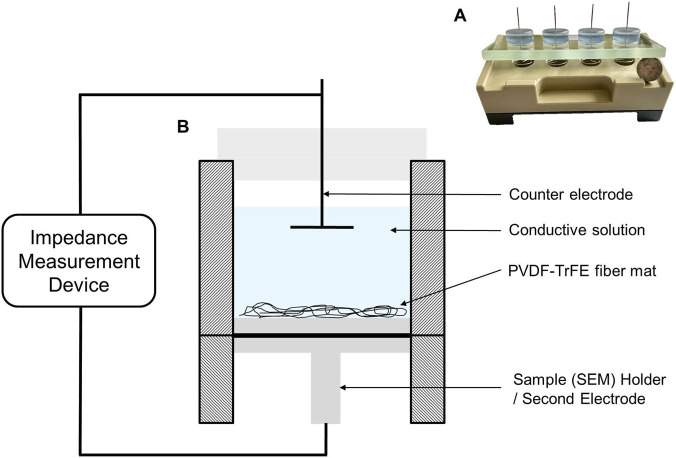
In-house-built measuring chamber **(A)** and schematic illustration of one of the four measuring cells **(B)**.

Fiber mats were again generated with three different spinning durations (15, 30, and 45 min). The thickness of the fiber mats varied with spinning time, increasing for longer spinning durations. Prior to the measurements, the fiber mat stripes were cut into 2 cm-wide, rectangular samples. Subsequently, the samples were pressed into the measuring cells of an in-house-built measuring chamber (Figure 1A) using a conventional SEM-Holder (12.7 mm in diameter).

Next, 1 mL of physiological saline solution (0.9 wt% NaCl; B. Braun) was added to each measuring cell and the platinum counter electrode inserted from above (Figure 1B). Impedances were measured with an electrochemical workstation (IM6eX, Zahner, Kronach, Germany) applying a sinusoidal current with an amplitude of 50 µA in a frequency range of 10 Hz–100 kHz. The amplitude of the current was chosen according to previous studies *in vitro* (Newbold et al., 2011) and in patients (Konrad et al., 2023).

Additionally, the development of electrical resistance during the wetting process of the hydrophobic fiber mats was monitored hourly, with readings taken for 24 and 72 consecutive hours on ten independent samples for each spinning duration. To further evaluate the influence of wetting, ten samples of each spinning duration were immersed in 2 L of physiological saline solution and incubated at 37 °C (Heracell VIOS 160i, Thermo Scientific) for 1, 2 and 3 weeks before electrical assessment. Reference values obtained from measurements of the SEM-Holder without fiber mat were subtracted from all values to account for background resistance.

### 2.4 Cell proliferation test

One of the main objectives of modifying the electrode surface was to reduce connective tissue growth. To simulate the behavior of cells directly growing on the fiber mat, an *in vitro* experiment with samples of the fiber mat and NIH/3T3 eGFP fibroblasts was conducted.

For the cell proliferation tests, circular samples (6 mm in diameter) were cut off the 30-min fiber mat stripe using a punching tool (Pfaffrath, Wiesbaden, Germany). The samples were sterilized with 70% ethanol as described above. Due to its hydrophobicity, floating of the fiber mat, once cell culture medium was added, had to be prevented. Therefore, high-vacuum grease (Dow Chemical, Wiesbaden, Germany) was disinfected by exposure to UV radiation for 30 min in a Spectrolinker XL-1000 (Biotec-Fischer, Reiskirchen, Germany) and applied in a thin film onto a Petri dish. The sterilized fiber mat was then dragged single-sided through the grease and attached to the center of the bottom of a Nunclon 4-well multidish (Thermo Fischer Scientific).

In each well, 2,000 fluorescence-active fibroblasts were seeded at the center of the well, and cell culture medium was added to a final volume of 0.75 mL. Subsequently, the plate was incubated at 37 °C and 5% CO_2_ (CB150, Binder). After 24 h of incubation, the plates were removed from the incubator, the lid of the plate was replaced by a sterilized (UV irradiation) ultrathin ALA MEA-Sheet (ALA Scientific Instruments, Farmingdale, United States) for microscopy and wells were imaged under a confocal laser scanning microscope (CLSM) (TCS SP8; Leica).

While imaging, cells were excited with a 488 nm laser and emission was detected at 509 nm. Brightfield (BF) images were simultaneously acquired to localize the fiber mat in each experimental well. To preserve cell viability, the CLSM was enclosed within an incubator that maintained the temperature at 37 °C throughout the recordings. Following image acquisition, the ALA MEA-Sheet was removed under sterile conditions, the original lid was replaced, and the plate was returned to the incubator.

This procedure was repeated after an additional 24 h of incubation subsequently for seven consecutive days to document cell distribution and cell growth. Cell culture medium was changed every 2 days. The experiment was independently repeated five times, with each plate consisting of two wells with fiber mat and two reference wells.

To analyze the recordings, ImageJ version 1.54d (National Health Institute, Maryland, United States) was used. First, the BF and fluorescence emission images were merged and the edge of the fiber mat outlined by drawing a circular region. Next, the BF was subtracted again and histograms representing the intensity of each pixel within the circular region (fiber mat) and across the entire well were generated. The intensity values of the fiber mat were then subtracted from those of the entire well to obtain the histogram corresponding to the area surrounding the fiber mat (outer circle).

Afterwards, the mean intensity per pixel was calculated for the three different regions - fiber mat, outer circle and entire well - over the course of the 7 days. To determine the area covered by cells, a threshold value for pixel intensity, ranging from 0 to 255, was defined. Therefore, reference images of wells with attached fiber mats containing only cell culture medium were used and analyzed. Since 95% of the pixels in the reference wells had intensity values below 23, this value was set as the threshold for cell indication.

Finally, the percentage of pixels with intensities above 23 on and around the fiber mat was calculated as a measure for cell covering.

### 2.5 Statistical analysis

Statistical analysis was performed using GraphPad Prism version 9.0.0 (GraphPad Software, Boston, United States). Statistical differences were determined using one-way or two-way analysis of variance (ANOVA) followed by Tukey’s honest significant difference test. In the following results, data are presented as mean ± standard error of mean (SEM). *p*-values below 0.05 were considered statistically significant.

## 3 Results

### 3.1 Electrospinning and morphological examination

A stable fiber jet without any visible drops or breaks in the polymer jet was formed during all electrospinning processes. The resulting fiber mats displayed homogenous fibers with diameters of 1.03 µm ± 0.18 µm, 1.07 µm ± 0.3 µm, and 0.97 µm ± 0.25 µm for spinning durations of 15, 30, and 45 min, respectively (mean diameter and standard deviation, n ≥ 41). The variation in electrospinning process duration resulted in layer thicknesses of 87.1 µm ± 12.4 µm, 147.24 µm ± 47.45 µm and 209.85 µm ± 25.35 µm with increasing process duration (n = 20).

### 3.2 Biocompatibility

#### 3.2.1 MTT

Absorbance at a wavelength of 570 nm was monitored in the MTT assay. According to the standard (International Organization for Standardization, 2009), the cytotoxicity threshold is defined at 70% of positive control cell viability.

**FIGURE 2:**
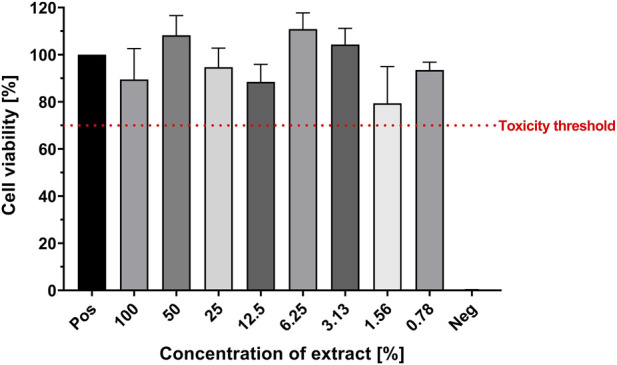
MTT assay results of the cell viability for the reference flask without interaction with the fiber mat. No significant difference between the positive control and any reference extract concentration was detected. The negative control (1:1 DMSO:DMEM) was significantly different from every other test group (*p* < 0.0001). N = 5 for all data points with n = 3 repetitions per plate.

For the reference solution generated by incubating and shaking the culture medium for 24 h, no cytotoxic effects or significant differences towards the positive control were observed for any concentration (Figure 2). Therefore, the preparation process of the extract solution is considered to have no impact on the integrity of the cell culture medium or the cell viability. Addition of DMSO as negative control resulted in no cell survival and was significantly different from every other experimental group across all three experiments.

**FIGURE 3:**
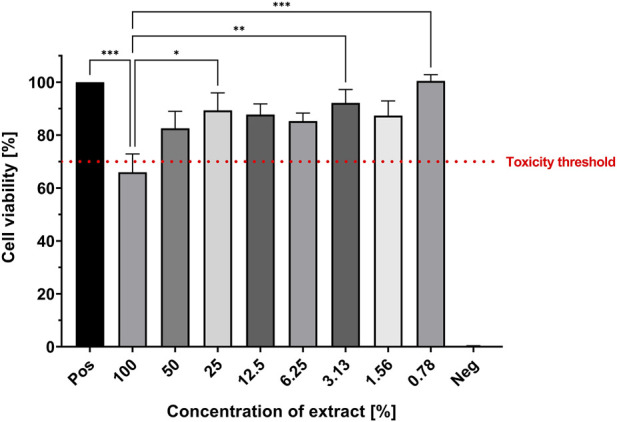
Cell viability after addition of the fiber mat extract solution with 4 h drying time after the sterilization procedure. *p* ≤ 0.05 (*), *p* ≤ 0.01 (**), and *p* ≤ 0.001 (***). The negative control showed a statistically significant difference compared to all other experimental groups (*p* < 0.0001). N = 6, n = 3.

Reduced cell viability was measured at 100% of the 4 h drying time fiber mat extract solution (66% ± 6.9%) (Figure 3). This reduction was significant compared to the positive control (*p* = 0.0002). The same applies to the 25% (89.3% ± 6.7%; *p* = 0.0296), 3.13% (92.2% ± 5.1%; *p* = 0.009), and 0.78% (100.5% ± 2.4%; *p* = 0.0002) extract solutions. All other concentrations showed neither a significant influence on the cell viability nor decreased beneath the toxicity threshold.

**FIGURE 4:**
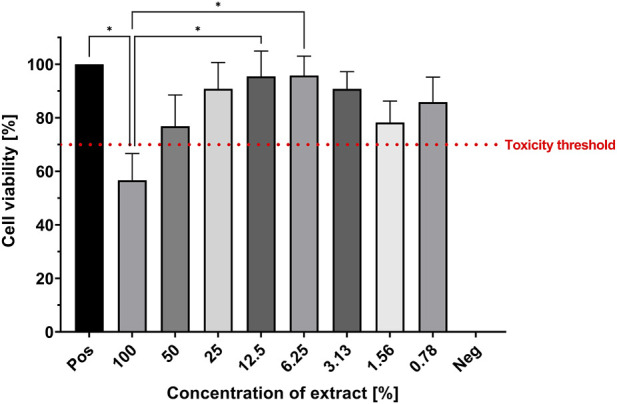
MTT assay results of the cell viability for the fiber mat extract solution (24 h drying duration). *p < 0.05. All concentrations of extract solution and the positive control were statistically different from the negative control (*p* ≤ 0.0006). N = 5, n = 3.

To assess whether the observed decrease in viability was attributable to ethanol residues remaining after the 4-h drying process, the experiments were repeated with the drying time increased to 24 h. Comparable results in the MTT assay were obtained (Figure 4). The cell viability for 100% of the extract solution was reduced to 56.6% ± 10% and significantly different from the positive control (*p* = 0.018) but not significantly different from 100% of the 4 h drying time (*p* = 0.6). Further significant reductions in cell viability compared to the positive control were observed for the 12.5% (95.5% ± 9.5%; *p* = 0.049) and the 6.25% (95.8% ± 7.2%; *p* = 0.045) groups. For all other concentrations, no values below the toxicity threshold were recorded and none were significantly different from the positive control. Therefore, the drying time of the fiber mat in the sterilization process did not influence the MTT assay results.

#### 3.2.2 SGN survival

**FIGURE 5:**
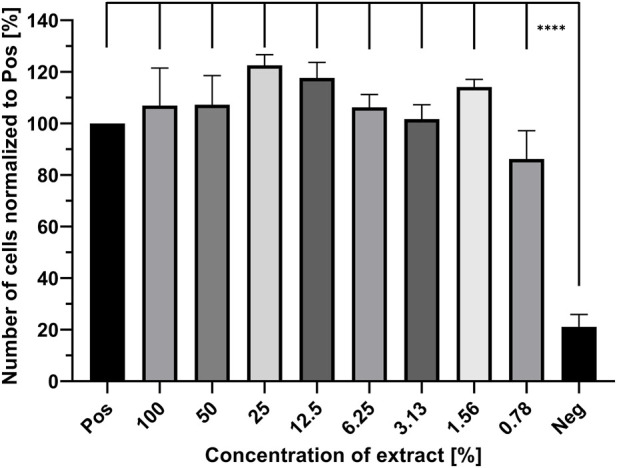
Percentage of neuronal survival normalized to the positive control over the concentration of extract solution (24 h drying time). ****p ≤ 0.0001. N = 5, n = 3.

Neuronal survival after exposure to the fiber mat extract solution was highest at a concentration of 25% (122.5% ± 4.1%) and was lowest at 0.78% (86.2% ± 11%) (Figure 5). Nonetheless, no significant difference to the positive control was detected for any concentration. Treatment with the known toxic 1:1 DMSO:complemented Panserin 401 solution (negative control) resulted in a decrease in neuronal survival (21.1% ± 4.8%) and differed significantly from all other data points (*p* ≤ 0.0001). The fiber mat extract solution did not have an impact on the number of surviving neurons.

#### 3.2.3 Neurite outgrowth

**FIGURE 6:**
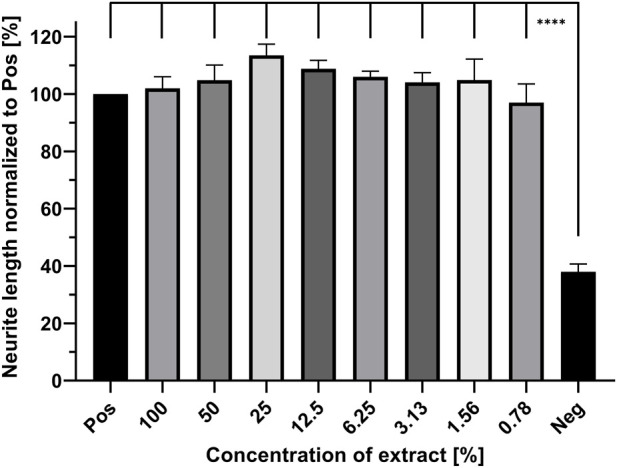
Percentage of neurite length over the concentration of the fiber mat extract solution (24 h of drying time) normalized to the positive control. ****p ≤ 0.0001. Experiments were independently conducted five times (N = 5, n = 3).

Similar effects were observed for the neurite length of the surviving neurons (Figure 6). In the positive control group, the average length of the longest neurites measured was 328.9 nm ± 32.7 nm. For the experimental groups, the largest mean value for neurite length was measured at 25% of the fiber mat extract solution (374.4 nm ± 41.4 nm; 113.5% ± 4% of the positive control), while the shortest mean value was recorded at 0.78% (316.1 nm ± 34.24 nm; 97% ± 6.5% of the positive control). Once again, the only significant difference (*p* < 0.0001) compared to the positive control and all other data groups was detected for the negative control with a mean neurite length of 123.2 nm ± 10.1 nm (38% ± 2.7% of the positive control). The extract solution did not alter the mean length of the longest neurites for all concentrations.

### 3.3 Electrical conductivity

**FIGURE 7:**
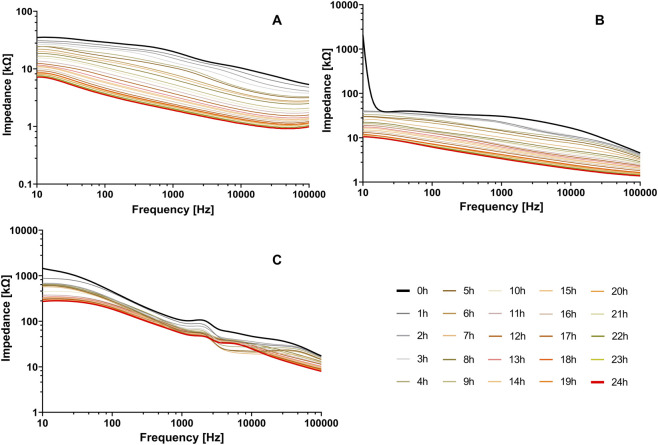
Mean impedances for the fiber mat samples in the measuring chamber over the frequency of stimulation. Impedances were measured hourly for 24 h **(A)** 15 min, **(B)** 30 min, and **(C)** 45 min of spinning time. For better visualization in **(A)** the y-axis is ranging from 0.1 to 100 Ω in contrast to **(B)** and **(C)**, where the range is 1–10,000 Ω. Each line represents one measurement and the black line indicates the starting value for each group and the last measurements after 24 h are marked in red. Sample size was N = 10 for the 15 min and 45 min groups and N = 9 for the 30 min group.

For the electrical analysis, impedance measurements were performed on the three fiber mats (15, 30, and 45 min spinning time), with readings recorded hourly over a 24-h period (Figure 7). Impedance decreased with increasing frequency across all three sample groups. Larger impedance drops were recorded at lower frequencies. For each spinning duration, impedance was highest at the beginning of the measurements and declined to a minimum after 24 h. One exception was found for the mats with the longest spinning time, where in the frequency range of 3.6 kHz–29.4 kHz, the measured mean frequency-dependent resistance showed irregularities in the pattern from hour one to hour eight (Figure 7C). After this deviation, the pattern of decreasing impedances over time resumed and continued until the end of the measurements. Larger impedance declines during the first hours of observation suggest that the wetting of the fiber mat progressed more rapidly in that time span. Nonetheless, continuing impedance decreases after 23 h of measurements indicated that wetting was not completed. In general, the impedance at each time point and frequency was higher with longer spinning durations of the fiber mat. The only exception was found for the impedance at 10 Hz for the 30-min spinning time (2.1 MΩ) compared to the 1.5 MΩ of the 45-min fiber mat (Figures 7B,C). However, after 1 hour this value returned to the standard pattern. Therefore, longer spinning times and thicker fiber mats resulted in higher electrical resistance.

**FIGURE 8:**
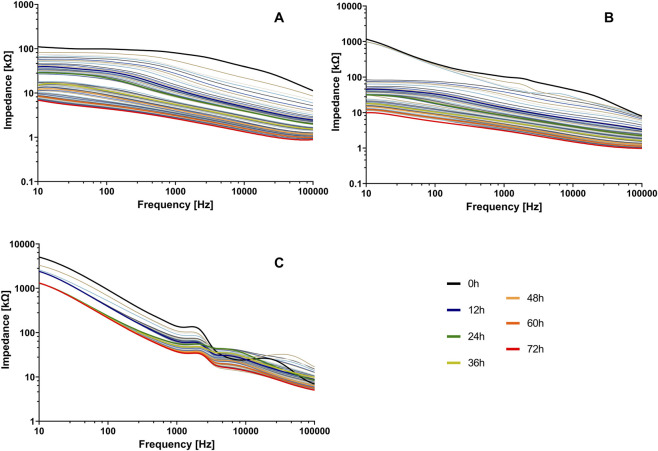
Mean impedance values across the stimulation frequency range for the fiber mat samples with **(A)** 15 min, **(B)** 30 min, and **(C)** 45 min of electrospinning time. Impedances were measured once per hour for 72 consecutive hours. To improve visualization, the y-axis range for each illustration was chosen individually. Each individual line represents one measurement. The black line indicates the starting impedance values for each group and the results after 72 h are marked in red. Intermediate curves at the time points of 12 (blue), 24 (green), 36 (yellow green), 48 (light orange), and 60 h (orange) are highlighted as well. Sample size was N = 10 for all three groups.

In the next step, further independent impedance measurements for 72 consecutive hours were performed to investigate wetting behavior of the fiber mat and its influence on the electrical conductivity (Figure 8). For the fiber mats generated by 15 min of electrospinning, results corresponded to the 24 h observations (Figure 8A). The starting impedance was highest, followed by larger impedance declines in the first 12 h and reaching the lowest values at 72 h. Additionally, impedances decreased with increasing frequencies. With the longer spinning time of 30 min, the impedances follow a similar pattern with in total larger values (Figure 8B). The only irregularities were detected from hour zero to hour three, where in the frequency range of 10–100 Hz only small differences in impedance were recorded. Additionally, at higher frequencies (around 4 kHz) the electrical resistance increased with increasing frequency (after 3 hours) or dropped inconsistent (1.5–2 kHz, after 2 hours). After this time, the curves stabilized and returned to the regular trend with the lowest measured impedance curve at 72 h. The last set of measurements was performed for the fiber mat spun for 45 min (Figure 8C). Comparable to the 30-min fiber mat samples, the curves for hour zero to hour three deviated from the rest of the hours with a sharp decrease in impedance beginning at 2 kHz and increases from 16.5 kHz to 34 kHz. The frequencies below 2000 Hz were not affected and showed a decline in resistance with increasing time. After 24 h, no change in impedance was measured for the value at 10 Hz. Moreover, in the frequency range of 10 Hz–1,000 Hz, impedance values decreased only slightly over time. Above 1 kHz, more irregularities were observed. As impedances first dropped until hour 12, then increased until hour 24 to subsequently decrease with increasing time. Nevertheless, and in accordance with the other sample groups, the lowest impedances were measured after 72 h. The results indicate that wetting of the fiber mat continues to progress after 24 and even 72 h, although changes in impedances become smaller at later time points.

**FIGURE 9:**
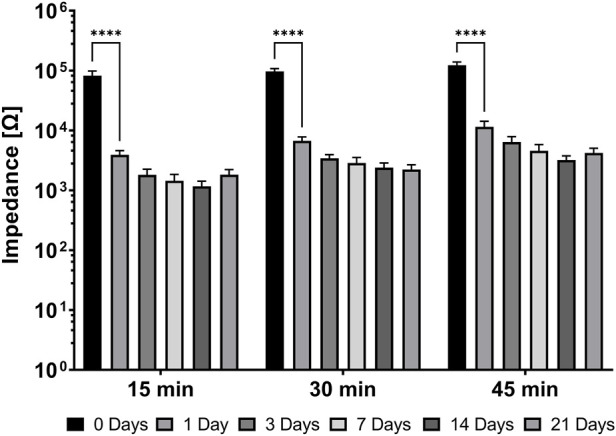
Changes in mean impedances at 1 kHz for the three different fiber mats over the course of 3 weeks. Only successive time points were statistically compared for all three spinning times. ****p ≤ 0.0001. N = 10.

In the last experiment, the long-term influence of wetting on impedance values was investigated by immersing fiber mat samples of all three spinning durations in sodium chloride solution for up to 3 weeks (Figure 9). Representative impedances at 1 kHz were compared to their respective preceding time points. The results are in coherence with the ones documented in the previous two analyses, where all three sample groups exhibited significant decreases in impedance (*p* < 0.0001), and primary wetting occurred in the first 24 h. Later, the impedance continued to decline, reaching its minimum at 14 days for the 15- and 45-min fiber mat samples (1.17 ± 0.25 kΩ and 3.21 ± 0.56 kΩ, respectively) and 21 days for the 30-min group (2.23 ± 0.44 kΩ). Nonetheless, these late changes in electrical conductivity were not statistically significant. The increases in impedances from time point 14 days of incubation to 21 days of incubation for the shortest (1.17 ± 0.25 kΩ to 1.82 ± 0.4 kΩ) and the longest (3.21 ± 0.56 kΩ to 4.21 ± 0.83 kΩ) spinning time fiber mat samples implied a stagnated wetting process after one to 2 weeks. At the state of complete wetting, the impedance increased approximately 1 kΩ for every additional 15 min of spinning time. Across all time points, measured frequency-dependent resistance remained highest for the thickest fiber mat (45 min) and lowest for the thinnest (15 min), consistent with the results of the earlier experiments.

### 3.4 Cell proliferation

**FIGURE 10:**
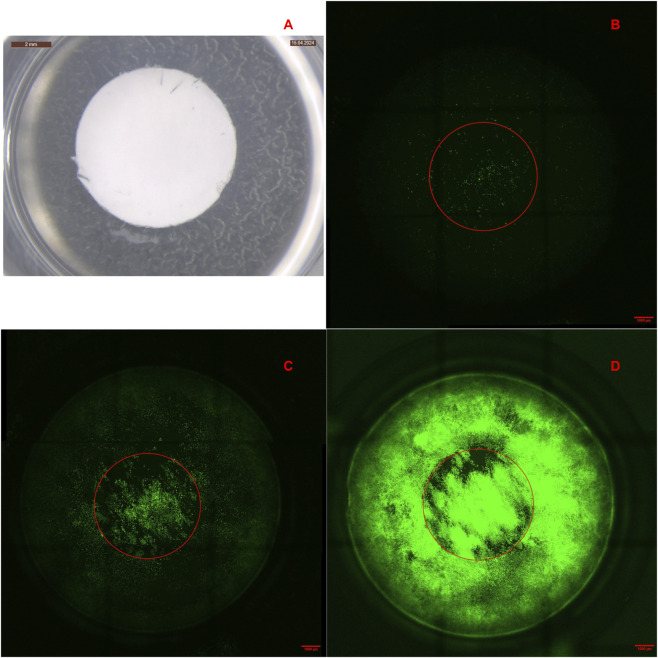
Sterilized and attached circular fiber mat sample in a well **(A)** (scale bar = 2 mm). CLSM images documenting the progression of cell growth after 1 day **(B)**, 4 days **(C)** and 7 days **(D)** of incubation (scale bar = 1 mm). The red circle indicates the fiber mat position in the well.

The fluorescence activity of the NIH/3T3 eGFP fibroblasts over a 7-day incubation period was documented using CLSM (Figure 10). Sterilization and attachment of the fiber mat to the bottom of the experimental well was successfully performed (Figure 10A). Figures 10B–D illustrates the progression of cell growth in one of the ten experimental wells at three time points. After the first 24 h, the images reveal an equal distribution of cells across the well (Figure 10B). After 4 days, cell proliferation could be observed. However, no visible differences between the fiber mat and the surrounding area (outer circle) were monitored (Figure 10C). After 7 days, differences between the fiber mat and the outer circle in the well became apparent (Figure 10D). More dark regions were recorded on the fiber mat than on the outer circle, indicating reduced cell coverage on the polymeric structure.

**FIGURE 11:**
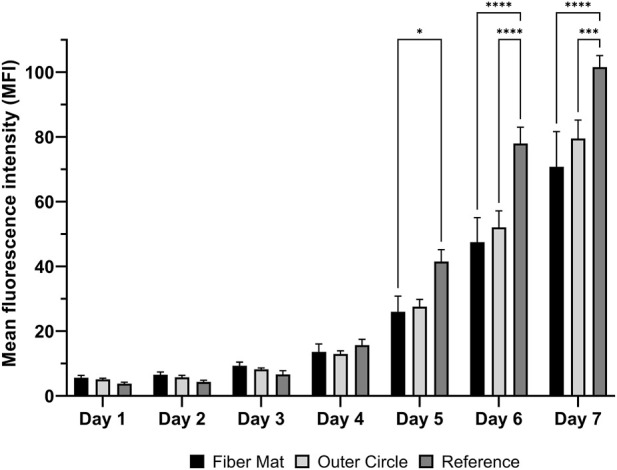
Mean fluorescence intensity of NIH/3T3 eGFP fibroblasts, measured over the course of 7 days for the reference (well containing cells without fiber mat), the outer circle (cell culture area surrounding the fiber mat) and the fiber mat itself. Background fluorescence from wells with medium and fiber mat but without cells was subtracted beforehand. *p < 0.05, ***p < 0.001, ****p ≤ 0.0001. N = 10.

To quantify the visual results, mean fluorescence intensity (MFI) expressed by fluorescence-active NIH/3T3 cells was calculated to determine cell proliferation behavior in direct contact to the fiber structure over the 7-day observation period (Figure 11). Within the first 4 days, the MFI on the fiber mat was greater compared to the outer circle and higher for 3 days compared to the reference well. Moreover, changes in MFI were limited during the first 3 days across all experimental groups (e.g., 5.58 ± 0.74 on day 1 to 9.28 ± 1.14 on day 3 for the fiber mat). The first significant differences in MFI between the fiber mat and the reference well appeared on day five (fiber mat = 25.96 ± 4.87, reference = 41.51 ± 3.65; *p* = 0.0265) and grew on day six (47.53 ± 7.53, 78.01 ± 5; *p* < 0.0001) and day seven (70.77 ± 10.87, 101.6 ± 3.54; *p* < 0.0001). Although MFI was higher for the outer circle compared to the fiber mat from day 5 (27.57 ± 2.22) onward, and the difference between them increased over time, a significant difference was not detected. The only significant difference for the outer circle was observed compared to the reference (*p* < 0.0001 day 6, *p* = 0.0008 day 7).

**FIGURE 12:**
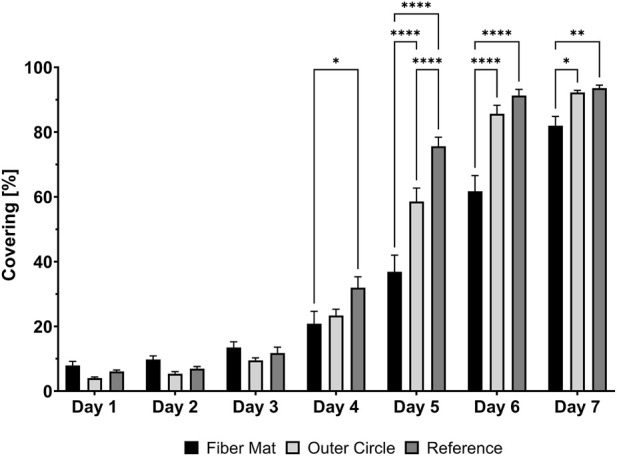
Percentage of cell coverage for the NIH/3T3 eGFP fibroblasts over 7 days of growth for the reference, the outer circle and the fiber mat. *p < 0.05, *p < 0.01, ****p ≤ 0.0001. N = 10.

To determine the area covered by fibroblasts, the percentage of pixel exceeding the threshold MFI value of 23 was calculated based on the MFI data obtained over the 7-day observation period (Figure 12). Similar to the MFI results, increases in cell covering were modest within the first 3 days, and the percentage was highest for the fiber mat although no significant differences among the three experimental groups were detected. From day four onward, the cell-covered area increased substantially and was significantly smaller on the fiber mat compared to the reference (*p* = 0.011). In contrast to the MFI results, on day five the cell covering on the outer circle became significantly higher compared to the fiber mat (*p* < 0.0001) and persisted until day seven (*p* = 0.013). Furthermore, the only significant difference between the outer circle and the reference was recorded for day five (*p* < 0.0001). On day six and seven, no significant difference between the outer circle and the reference well was recorded and by the last day, cell covering almost reached 100% for both groups (92.2% ± 0.7% outer circle, 93.6% ± 0.9% reference) while the fiber mat remained significantly lower at 82% ± 2.9% (*p* = 0.0073 towards the reference).

The results of both observations (Figures 11, 12) indicated that cell proliferation is reduced on the fiber mat as compared to the regular cell culture material.

## 4 Discussion

The CI electrode array is intended to stimulate auditory neurons to restore the hearing ability in patients. However, trauma induced during electrode insertion, together with the subsequent foreign body response, results in encapsulation of the implant and increased electrical impedances (Konrad et al., 2023). Elevated electrical resistances adversely affect the CI performance leading to a reduction in hearing quality for the patient (Newbold et al., 2014). Several approaches to attenuate inflammatory responses or enhance electrical conductivity have been conducted, including surface patterning of the electrodes or surface modification with drug-loaded polymer coatings (Linke et al., 2015; Xu et al., 2018; Luo et al., 2021; Wulf et al., 2022). Nonetheless, up to date, uncontrolled intraoperative administration of steroids into the cochlea (Paasche et al., 2009), release of dexamethasone incorporated into the silicone of the electrode array (Simoni et al., 2020; Rongthong et al., 2022), and the use of an inner ear catheter for direct drug administration (Dhanasingh and Hochmair, 2021; Tuset et al., 2025) have demonstrated a sustained reduction in connective tissue formation and preservation of residual hearing over several years. Modifying the surface with an additional layer of porous fiber mats composed of hydrophobic PVDF-TrFE may present a promising alternative to mitigate inflammatory effects without administration of pharmacological agents.

During the electrospinning process, the relative humidity ranged from 30% to 69%. For hydrophobic polymers, previous studies have reported that elevated humidity levels can lead to increased fiber diameters and bead formation. This effect is attributed to water absorption into the polymer jet, which promotes accelerated solidification due to precipitation, resulting in reduced elongation during the flight phase of the polymer jet (Mailley et al., 2021). It cannot be excluded that the described effect influenced fiber size in the current study, however, due to the comparable average fiber diameters received for all spinning durations in this study, humidity did not exert a dominant effect on results.

The biocompatibility results indicate that extracts derived from the micro-networks are biocompatible, with a single restriction observed at 100% extract concentration in the MTT assay, where cell viability decreased below the toxicity threshold of 70%. Two different drying times following fiber mat sterilization were evaluated to determine whether residual ethanol from the sterilization process influenced the biocompatibility outcomes (Figures 3, 4). As both assays exhibited comparable cell viability results, the extended drying time was considered irrelevant for the results of the biocompatibility test. The solvents used for polymer dissolution (acetone and DMF) may have remained within the fiber mats after electrospinning and potentially affected cell viability, as these solvents have shown cytotoxic potential even in lower concentrations (Gu and DeAngelis, 2005). However, the absence of inhibitory effects on neuronal survival or neurite outgrowth suggests that residual solvents were not responsible for the reduced fibroblast viability.

Alternatively, Chavoshnejad et al. described that the mechanical behavior of electrospun fiber mats strongly depends on the degree of bonding between the fibers. Without significant bonding, fibers can slide and separate under mechanical stress (Chavoshnejad and Razavi, 2020). Therefore, cutting the fiber mats into samples and shaking them for 24 h to prepare the extract solution may have disrupted these fiber bonds, resulting in the release of loose fibers into the culture medium. When the fibroblasts were exposed to 100% of the extract, direct contact with these fibers may have impaired cell proliferation. Consequently, fewer cells were available to metabolize MTT into formazan crystals compared to the positive control and lower extract concentrations, resulting in a reduction in measured cell viability. In addition, Gryshkov et al. have reported reduced metabolic activity in WST-1 assays but found no impairment of cell viability when analyzing fiber-mat–attached cells using immunofluorescence (Gryshkov et al., 2021). This explanation could also explain the discrepancy between MTT assay and neuronal survival, as neurons are not affected by proliferation inhibition and therefore exhibited no signs of cytotoxicity at any extract concentration. Based on this interpretation and the overall biocompatibility results, the fiber mats are considered biocompatible. Furthermore, Bryan et al. have observed no decrease in cell viability when using electrospun PVDF-TrFE scaffolds and have even shown enhanced cell viability through ultrasound stimulation of the fiber mat (Bryan et al., 2023).

Although PVDF-TrFE is a widely used material for biomedical applications, its influence on electrical resistance, when applied as an additional surface layer, has not yet been evaluated. Existing applications primarily focus on the piezoelectric properties of the material, whereby it generates electrical signals in response to mechanical deformation (Park et al., 2018; Orkwis et al., 2020; Slobodian et al., 2022). Its hydrophobic and biocompatible nature, combined with its suitability for electrospinning, renders PVDF-TrFE a promising candidate for the approach to reduce connective tissue growth on the CI electrodes through surface modification with fine micro-networks.

Wulf et al. demonstrated that coating stimulation electrodes with PLLA increases the impedance to an extent that reductions in biological insulation by connective tissue may become negligible (Wulf et al., 2022). They further detected substantial impedance variations depending on the coating. Coatings exhibiting cracks or imperfection allowed the conductive solution to penetrate the additional layer and establish contact with the electrode surface over time. These findings suggest that porous structures, such as fiber mats generated by electrospinning, may exert a limited influence on electrical resistance while simultaneously reducing biological insulation, potentially resulting in an overall impedance reduction compared with conventional implants.

Fiber mats are hydrophobic structures containing trapped gases within their pores. The wetting behavior depends on both the layer thickness and pore size (Altschuh et al., 2022). The wetting process can be divided into three different stages: non-wetted, partially wetted, and fully wetted (Chenoufi et al., 2026). The transition between these stages can be accelerated by addition of surfactants or by controlled changes in atmospheric pressure (Bagrov et al., 2021; Chenoufi et al., 2026). However, even without any external factors, wetting progresses with gradual invasion of liquid into the pore network, driven by capillary forces and surface energy minimization (Gryta and Barancewicz, 2010). As the partial pore filling advances, electrical resistance decreases until reaching its saturation point upon complete wetting. This progressive wetting procedure of hydrophobic fiber mats in saline solution could explain the wetting results observed in this study.

The impedance measurements of deposited fiber mats on conventional SEM-holders exhibit a decrease in electrical resistance as the wetting process of the fiber mat progresses. These results are consistent with those reported by Sharma et al., where impedance values for hydrophobic polyvinyl alcohol (PVA) fiber mats decreased from 1.2 × 10^8^ Ω to 584 Ω following coatings that increased hydrophilicity and thereby wetting of the fiber mat (Sharma et al., 2023). In the current study, once completely wetted, impedance values of approximately 1–3 kΩ at 1 kHz were observed, depending on fiber mat thickness. According to Ohm’s law, electrical resistance depends on the surface area of the electrodes. The surface of the SEM-holders used in this study is considerably larger than the one of CI electrodes (126.6 mm^2^ versus 0.38 mm^2^ (Newbold et al., 2004)). Therefore, although the impedance results are promising, they cannot be directly compared with impedance values obtained from non-coated CI electrodes. Furthermore, due to the large difference in surface area, the observed linear increase in impedance of approximately 1 kΩ for every additional 15 min of spinning time on the SEM holder cannot be directly extrapolated to the electrodes of the implant but needs to be investigated. Other studies investigating non-porous PLLA coatings of varying thicknesses on stimulation electrodes have reported initial impedances exceeding 1 MΩ (Wulf et al., 2022). Over time, particularly in coatings exhibiting structural imperfections or cracks, impedance values for several electrodes decreased to below 10 kΩ. These findings suggest that, if complete wetting occurs, similar effects and even lower impedance values may be achievable for the investigated porous fiber mats on stimulation electrodes as the accumulated pore area is expected to be larger than the open cross-section of the cracks in the published study. Nevertheless, impedances on CI electrode contacts should be below 3–4 kΩ after electrospinning as impedances in CI patients can be in this range even years after implantation (Konrad et al., 2023).

Irregularities in the trend of decreasing impedances with increasing wetting time were observed for fiber mats produced with longer spinning durations. Sudden impedance drops at lower frequencies for the 45-min fiber mat between consecutive measurement intervals suggest that the wetting process was less linear than for the other two fiber mats. The increased complexity of the porous structure with greater thickness may have impaired the uniform progression of the conductive solution through the fiber mat, resulting in intermittent, pronounced reductions in impedance. At higher frequencies, impedance values initially decreased during the first 12 h and subsequently increased over the following 12 h, for which only tentative explanations can be proposed. For longer spinning durations more material is used to generate the fiber mat leading to more contribution of the piezoelectric effect of the PVDF-TrFE. When this property is electrically stimulated more frequently, repeated mechanical elongation and compression results. Such mechanical dynamics may alter the fiber mat microstructure during the initial measurement period, causing deviations in electrical conductivity. Once structural stabilization is reached, the system returns to a consistent trend of decreasing impedance over time. This behavior was observed for both measuring periods (24 and 72 h), albeit with different stabilization times, indicating that it is a characteristic of the fiber mat architecture.

Cell proliferation was assessed only for the 30-min fiber mat. Previous studies investigating the influence of femtosecond laser–microstructured platinum surfaces on cell behavior have demonstrated that growth of NIH/3T3 fibroblasts is primarily affected by microstructures in the width range of 4–7 µm (Reich et al., 2008). Furthermore, Aliuos et al. have reported that groove depths in titanium laser-microstructed surfaces of 10 µm or more did not result in significant differences in cell proliferation, indicating no effect of increased microstructure depth on cell proliferation (Aliuos et al., 2013). Moreover, it is reported that rat dermal fibroblasts do not really “see” the bottom of microgrooves if the depth is larger than 1 µm (Walboomers et al., 1999). All three fiber mats used in this study exhibited thicknesses larger than 80 µm. Therefore, variations in thickness were considered negligible with respect to cell proliferation as from the cell’s perspective, the three different mats were indiscernible.

Other studies have documented the use of eGFP-expressing NIH/3T3 fibroblasts as a non-invasive method to quantify cell proliferation, as fluorescence intensity correlates with the cell number (Zhang and Yang, 2011; Miller et al., 2017). As the fluorescence signal of these cells can be used instead of a metabolic assay (Miller et al., 2017), we assume that MFI is not influenced by cell cycle or metabolic activity as the fiber mats were proven to be biocompatible. The different surface morphology of the fiber mat compared to the smooth surface of the cell culture well could potentially exert some substrate-induced stress on the cells. This might influence gene activity and, therefore, could have an effect on GFP expression in the cells. However, the development of MFI or surface covering over time should not be affected.

While other studies have employed electrospun PVDF-TrFE fiber mats to promote cell proliferation and alignment by harnessing the piezoelectric properties of the material (Wang et al., 2018; Orkwis et al., 2020; Bryan et al., 2023), the primary objective in the present study was to investigate potential reduction of connective tissue growth on the implant surface using the fiber mat coating without exploiting the piezoelectric effect. The results demonstrated that fibroblast proliferation was reduced on the fiber mat compared with the standard cell culture material, although this reduction was statistically significant only for cell covering and not for the MFI. For the first 3 days, both MFI and cell covering values were highest on fiber mats. One possible explanation for this observation is the initial cell seeding location within the wells. Cells were seeded at the center of each well across all samples, which coincided with the placement of the fiber mats. Consequently, a higher number of cells initially settled directly onto the fiber mats and adhered to their surface. Given that the fiber mats reduce cell proliferation, the initially higher cell density and fluorescence intensity persisted only for first 3–4 days, until cell growth on the standard culture material surpassed the initial imbalance caused by the seeding location. After 6 days of cultivation, the difference in cell covering between the fiber mats and the outer circle reached a maximum, with 24% greater covering on the cell culture material. By day seven, this difference decreased to 10.3%, primarily because cell covering on the outer circle approached saturation at nearly 100%, while cell proliferation on the fiber mats continued. Therefore, it can be assumed that, with extended observation time, cell covering on the fiber mat would also reach 100%. These findings imply that the micro-structured fiber mat surface reduces and delays fibroblast coverage but does not completely inhibit it.

Cell morphology and cell adhesion strength are strongly influenced by the surface properties of the cell culture substrate. In this study, GFP-expressing NIH/3T3 fibroblasts were used to quantify general proliferation behavior on the fiber mat and to compare cellular responses between a conventional cell culture substrate and the porous structure. Further investigations regarding the influence of the fiber mat properties such as fiber diameter or pore size on the cell proliferation have to be conducted. In addition to proliferation analysis, previous studies have demonstrated that GFP fluorescence can also be employed to document changes in cell morphology depending on the surface topography (Litman et al., 2000; Aliuos et al., 2013). Atomic force microscopy (AFM) is frequently used to characterize the topography of surfaces. Moreover, AFM provides the possibility to investigate cell adhesion via single-cell force spectroscopy, where individual cells are detached from the substrate and the required detachment force is recorded (Helenius et al., 2008; Aliuos et al., 2013; Friedrichs et al., 2013). Therefore, investigations using AFM could contribute to deeper understanding of the cell behavior on the fiber mats.

In this study, first *in vitro* insights into fibroblast responses to electrospun PVDF-TrFE fiber mats were obtained and analyzed to assess their general potential to reduce the electrical insulation due to cellular overgrowth. However, the foreign body response *in vivo* is considerably more complex, involving distinct, time dependent stages such as protein adsorption, inflammation and fibrosis (Saleh and Bryant, 2017). Some of these influences such as the influence of protein adsorption or even biofouling processes could be investigated *in vitro* in future studies whereas others such as tissue response in a natural environment finally have to be investigated *in vivo*.

## 5 Conclusion

In this study, PVDF-TrFE fiber mats fabricated by electrospinning were evaluated with respect to their biocompatibility, electrical conductivity, and influence on cell proliferation on their surface to determine their suitability as a surface modification of CI stimulation electrodes. The fiber mats were biocompatible. Once fully wetted after one to 2 weeks, the increase in impedance measured on SEM-holders was modest. Cell proliferation was reduced but not inhibited, resulting in delayed cell covering as compared with the standard cell culture substrates. Further investigations are required to quantify the impedance changes after applying the fiber mats directly to CI stimulation electrodes and to develop strategies to accelerate the wetting process.

## Data Availability

The datasets presented in this study can be found in online repositories. The names of the repository/repositories and accession number(s) can be found below: 10.6084/m9.figshare.31095871.
